# A salient effect of density on the dynamics of nonaqueous electrolytes

**DOI:** 10.1038/srep46718

**Published:** 2017-04-24

**Authors:** Sungho Han

**Affiliations:** 1 CAE Group, Platform Technology Lab, Samsung Advanced Institute of Technology, Suwon, Gyeonggi 16678, Korea

## Abstract

The mobility and solvation of lithium ions in electrolytes are crucial for the performance and safety of lithium ion batteries. It has been known that a single type of solvent cannot satisfy the requirements of both mobility and solvation simultaneously for electrolytes. Therefore, complex solvent mixtures have been used to optimize both properties. Here we present the effects of density on the dynamics and solvation of organic liquid electrolytes via extensive molecular dynamics simulations. Our study finds that a small variation in density can induce a significant effect on the mobility of electrolytes but does not influence the solvation structure of a lithium ion. It turns out that an adjustment of the density of electrolytes could provide a more effective way to enhance mobility than a control of the solvent mixture ratio of electrolytes. Our study reveals that the density change of electrolytes mainly affects the residence time of solvents in the first solvation shell of a lithium ion rather than the structural change of the solvation sheath. Finally, our results suggest an intriguing point for understanding and designing electrolytes of lithium ion batteries for better performance and safety.

As technologies and markets of portable electronic devices and electric vehicles are rapidly growing in recent years, rechargeable batteries such as lithium ion batteries have become one of the most active research fields and industrial markets^1^,^2^,^3^,^4^,^5^. Among components of a battery, electrolytes play a central role in the performance and safety of lithium ion batteries^1^,^2^,^4^,^5^,^6^,^7^,^8^,^9^,^10^. They allow for lithium ions to conduct between cathode and anode of batteries and contribute to form a solid electrolyte interphase (SEI) which is a key element for protection of electrodes from degradation^6^,^7^,^8^,^9^,^10^,^11^,^12^.

Ionic conductivity *λ* is one of main properties characterizing electrolytes, which quantifies how mobile the ions are for the electrochemical reactions^13^. Factors determining the ionic conductivity are the number of ions *n*^ion^, the magnitude of charge *Q*^ion^ which ions carry, and the mobility of ions *μ*^ion^, that is, 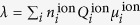
1. For given ions, therefore, a strategy to increase the ionic conductivity essentially includes improvements of both diffusivity and the number of ions participating in carrying charges^14^. Whereas larger diffusivity of ions obviously increases the ionic conductivity, forming a pair of a cation and an anion does not contribute the ionic conductivity due to its charge neutrality. In fact, the formation of pairs of cations and anions is closely related with a decrease in diffusivity due to an increasing size of ionic clusters in addition to a decrease in the number of ions contributing the ionic conductivity. Therefore, the pair formation is eventually connected with the reduction of the ionic conductivity. To hinder cations and anions from forming pairs and even clusters, it is required the solvation process of cations by solvents. It is generally expected that solvents in electrolytes should simultaneously both enhance the mobility of ions and form a proper solvation shell of cations.

A molecule with a large dielectric constant can fulfill a good solvent in the view of the ion pairing, but it is easy to fail to enhance the mobility of ions due to its large viscosity. In contrast, a molecule with a small dielectric constant has lower viscosity in order to enhance mobility, but its fulfillment in the solvation process is not satisfied. Instead of a single type of solvent, therefore, state-of-the-art electrolytes adopted in current lithium ion batteries consist of multiple types of solvents to compromise both properties: the mobility and the ion-pairing^1^,^5^,^15^. For example, ethylene carbonate (EC) has a large dielectric constant (*ε* ~ 90 at 40 °C) which is even higher than water (*ε* ~ 79 at 25 °C)^1^,^16^. However, its high viscosity (*η* ~ 1.9 cP at 40 °C) in addition to the high melting temperature (*T*_m_ ~ 36.4 °C) prohibit it from being chosen as a sole solvent. Dimethyl carbonate (DMC) has the low viscosity (*η* ~ 0.59 cP at 20 °C) but a small dielectric constant (*ε* ~ 3.1 at 25 °C). Therefore, a combination of cyclic and linear carbonates such as EC and DMC has been suggested as a candidate of efficient electrolytes to be satisfied with two important properties^1^,^17^,^18^.

In this work, we explore the effects of density on the dynamics of an electrolyte consisting of a lithium hexafluorophosphaste (LiPF_6_) salt in a binary solvent mixture of EC and DMC with a mixture ratio of EC:DMC = 50%:50% (in volume %). Note that for simplicity we will denote the solvent mixture ratio of electrolytes as only the EC ratio throughout this work. For comparison, we also investigate the dynamics for a case of EC 20%.

## Results

Our starting point is the two electrolyte systems with densities of *ρ* = 1.3446 g/cm^3^ for EC 50% and *ρ* = 1.2677 g/cm^3^ for EC 20%, and then we investigate the dynamics for EC 50% as a function of *ρ*. Those initial densities correspond to the total densities of binary mixtures of EC and DMC with 1 M LiPF_6_ when two systems have the same volume without considering a mixing effect of EC and DMC. Generally, the total density of a mixed system does not follow a simple summation: *ρ*_total_ ≠ *ρ*_simple_ = (*ρ*_EC_*V*_EC_ + *ρ*_DMC_*V*_DMC_)/(*V*_EC_ + *V*_DMC_), but the effect of the mixing should be included: *ρ*_total_ = *ρ*_simple_ + *ρ*_mixed_. The term *ρ*_mixed_ is generated by the interaction between EC and DMC, and it is difficult for *ρ*_mixed_ to be quantified. If one considers the mixing of EC and DMC, the total density will be different from one without it^19^. For example, the experimental density of the bulk electrolyte for EC 50% with 1 M LiPF_6_ at ambient conditions is known to be around *ρ* = 1.30 g/cm^3^ 20,21. Further, we consider five more densities of *ρ* = 1.3219, 1.3028, 1.2852, 1.2709, 1.2568 g/cm^3^ for the system of EC 50% to investigate how density can affect dynamical properties of electrolytes. Note that this is different from many studies of the salt effects on the dynamics of electrolytes, since in our study the initial salt concentration is fixed but the volume of the system is changed.

### Dynamics

**Figure 1:**
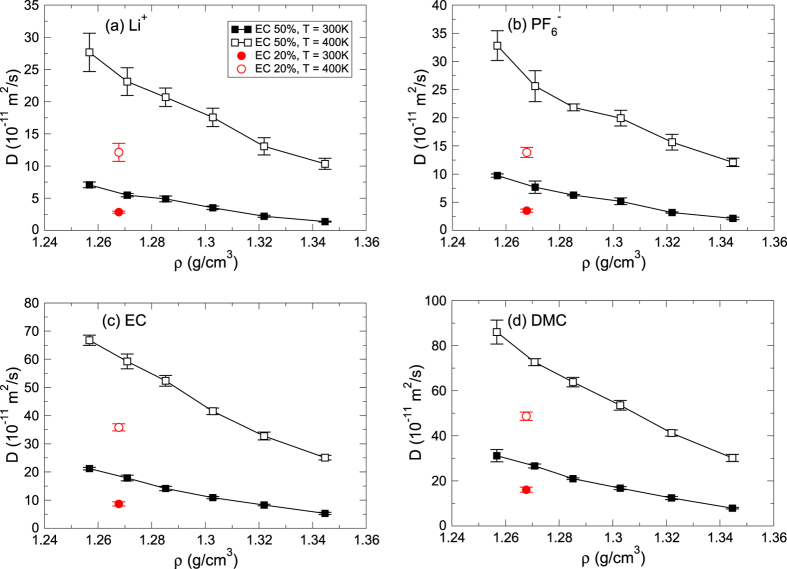
Diffusivity of an electrolyte. Shown are diffusion constants *D* of each component of an electrolyte, (**a**) a Li^+^ ion, (**b**) a PF_6_^−^ ion, (**c**) EC and (**d**) DMC, as a function of density *ρ* at temperatures of *T* = 300 K and 400 K for a solvent mixture ratio of EC 50%. For comparison, we also present the diffusion constant *D* of each component of an electrolyte for a solvent mixture ratio of EC 20% at a density of *ρ* = 1.2677 g/cm^3^. The results show that *D* exhibits the substantial dependence of *ρ* at a fixed mixture ratio of solvents. For both cation and anion, *D* for EC 20% shows a comparable magnitude with *D* at *ρ* = 1.3219 g/cm^3^ of EC 50% at both temperatures of *T* = 300 K and 400 K.

To study how the mobility of electrolytes is affected by density *ρ*, we first consider the diffusion constant *D* using the Einstein relation which is characterized by the mean squared displacement (MSD), defined as^22^,^23^


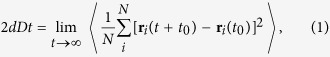


where *d* is the dimensionality of the system and 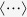 represents an ensemble average. In Fig. 1, we calculate *D* of each component of the electrolyte as a function of *ρ* for EC 50%. For all components, *D* is very sensitive to *ρ* compared with other liquid systems^22^. When *ρ* decreases by Δ*ρ* = 0.0878 g/cm^3^ from *ρ* = 1.3446 g/cm^3^ to 1.2568 g/cm^3^, *D* of a Li^+^ ion shows increases by a factor of 5.140 and 2.672 at *T* = 300 K and 400 K, respectively. We also observe the similar increases in *D* for the other components: 4.554 and 2.715 for an PF_6_^−^ ion, 4.007 and 2.661 for EC, and 3.959 and 2.853 for DMC at *T* = 300 K and 400 K, respectively. This implies that a small variation in density can induce a large impact on the diffusivity of electrolytes. As *T* increases, the effect of *ρ* on *D* becomes weaker.

It is interesting that *ρ* has the strong sensitivity of *D*. For liquid acetonitrile, for example, an experimental study showed that the decrease in *ρ* by approximately Δ*ρ* = 0.1 g/cm^3^ is desired to increase *D* by a factor of two at *T* = 298 K^24^. For water, it is showed that the decrease in *ρ* by approximately Δ*ρ* = 0.2 g/cm^3^ is desired to increase *D* by a factor of two at *T* = 300 K^22^. For organic liquid electrolytes, our results show up to a fivefold increase in *D* when *ρ* decreases by less than 0.1 g/cm^3^ at *T* = 300 K. It is surprising that *D* rapidly changes with the relatively small modification of *ρ*. Furthermore, *D* for EC 20% at *ρ* = 1.2677 g/cm^3^ shows a comparable magnitude of *D* for EC 50% at *ρ* = 1.3219 g/cm^3^. Thus our results indicate that in order to enhance *D* an adjustment of *ρ* could be a better strategy than a decrease in the EC fraction. The latter is known to be a conventional method accepted to increase the diffusivity (or decrease the viscosity) of electrolytes. In our results, the small change in *ρ* such as Δ*ρ* from *ρ* = 1.3446 g/cm^3^ to 1.3219 g/cm^3^ shows the larger increase in *D* of a Li^+^ ion than the change of the EC fraction from 50% to 20%. This situation is similar for the other components and the higher temperature. Note that the small change in density actually requires a large amount of change in pressure. In our case, pressures range from less than 1 MPa up to a few hundreds of MPa in accord with *ρ*. For liquid acetonitrile, the same pressure range has been experimentally examined and the rate of change in *D* is much larger in our case than liquid acetonitrile^24^.

**Figure 2:**
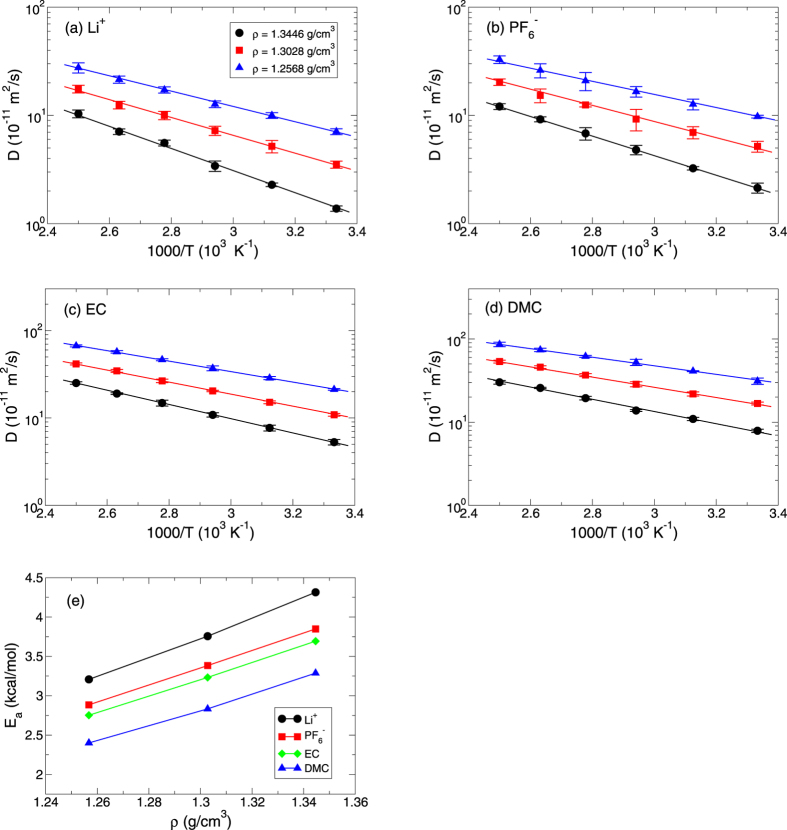
Temperature dependence of diffusion constants. Shown in an Arrhenius plot are diffusion constants *D* of each components of an electrolyte, (**a**) a Li^+^ ion, (**b**) a PF_6_^−^ ion, (**c**) EC and (**d**) DMC, for EC 50% at three densities of *ρ* = 1.2568, 1.3028, and 1.3446 g/cm^3^. All data are well fitted into an Arrhenius form, 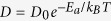. The results show that the slope of the fit increases as *ρ* increases. Solid lines are guides for eyes. (**e**) Activation energies *E*_*a*_ for diffusion of a Li^+^ ion, a PF_6_^−^ ion, EC and DMC as a function of density *ρ* for EC 50%, which is calculated from the slope of the Arrhenius plot. Clearly, it shows that *E*_*a*_ for all components of an electrolyte decreases as *ρ* decreases.

To see how *ρ* affects the activation barrier for diffusion, we now examine the temperature dependence of *D* for all components of an electrolyte for three different densities^19^, as shown in Fig. 2. In an Arrhenius plot, *D* is well fitted into an Arrhenius form, *D* = *D*_0_exp(−*E*_*a*_/*k*_*B*_*T*), where *D*_0_ is a pre-factor and *k*_*B*_ is the Boltzmann constant. We find that the absolute magnitude of the slope of the fitted line decreases upon decreasing *ρ*. In Fig. 2(e), we calculate the activation energy *E*_*a*_ for diffusion from the Arrhenius temperature dependence of *D*^21^,^25^. Our results show that *E*_*a*_ at *ρ* = 1.3446 g/cm^3^ is significantly larger than *E*_*a*_ at *ρ* = 1.2568 g/cm^3^. The ratio *γ* of *E*_*a*_ at *ρ* = 1.3446 g/cm^3^ to *E*_*a*_ at *ρ* = 1.2568 g/cm^3^ gives approximately *γ* = 1.34 for a Li^+^ ion, 1.33 for a PF_6_^−^ ion, 1.34 for EC and 1.37 for DMC, respectively. It seems that *E*_*a*_ increases with a similar rate for all components of the electrolyte as *ρ* increases. Our results show that the decrease in *ρ* results in the significant reduction of *E*_*a*_ for diffusion. Note that the magnitudes of *E*_*a*_ for all components show Li^+^ > PF_6_^−^ > EC > DMC, and it explains why DMC is the fastest component and a Li^+^ ion is the slowest^13^.

In the description of self-diffusion, Zwanzig interpreted diffusion as crossing an energy barrier from one local energy minimum to one of other local energy minima in the energy landscape over the whole phase space^26^. The energy landscape of the system is newly generated at each moment by updated coordinates and momenta of the systems. In the view of the energy landscape, a decrease in *ρ* can reduce the energy barrier between local energy minima, so that diffusion can be enhanced. As *T* increases, the effect of *ρ* will be reduced because the thermal energy becomes large enough for the barrier crossing. Our results are in good agreement with the Zwanzig’s interpretation of diffusion.

**Figure 3:**
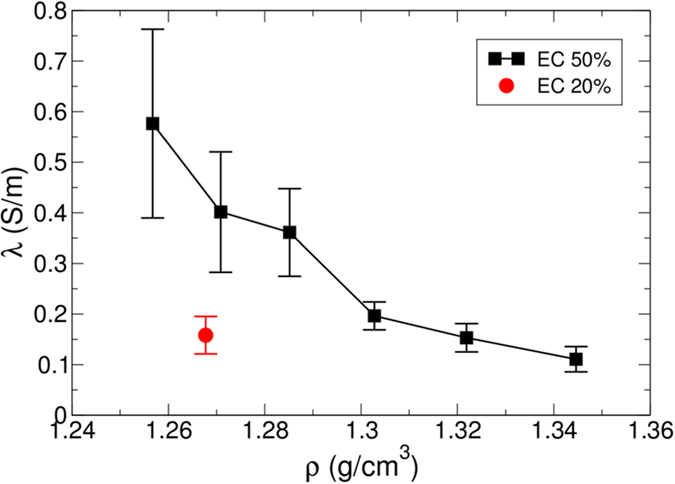
Ionic conductivity. Shown in the plot as a function of density *ρ* is the ionic conductivity *λ* at temperature *T* = 300 K for a solvent mixture ratio of EC 50%. For comparison, we also present *λ* for EC 20%. Similar to the diffusion constant *D, λ* shows the substantial dependence of *ρ. λ* for EC 20% is similar to *λ* at *ρ* = 1.3219 g/cm^3^ for EC 50%.

In addition to *D*, we calculate the ionic conductivity *λ* defined as^13^,^17^,^27^,^28^


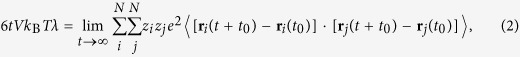


where *z* is the charge of an ion in the unit of the elementary charge *e* and 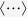 represents an ensemble average. The summations are over all ions of the system. As shown in Fig. 3, *λ* for EC 50% substantially increases upon decreasing *ρ*. When *ρ* is lowered to *ρ* = 1.2568 g/cm^3^ from 1.3446 g/cm^3^, *λ* increases by a factor of almost five which is the same amount as in *D*. Combined with the results of *D*, it is interesting that *λ* also exhibits the strong sensitivity on *ρ*. We also find that when *ρ* becomes 1.3028 g/cm^3^, *λ* for EC 50% shows the similar magnitude to one for EC 20%. Due to the competition between the mobility and ion pairing, it is known that the optimal solvent fraction of EC to give a maximum in *λ* is located between 20% and 30%^1^. Our results indicate that there is an alternative way to enhance *λ* without modifying the solvent mixture ratio of electrolytes. Namely, the adjustment of *ρ* provides more dramatic effects on *D* and *λ* than the modification of the solvent mixture ratio of electrolytes. Presumably, the rapid increase of *D* upon decreasing *ρ* gives rise to the unexpected sensitivity of *λ* on *ρ*.

Now the remaining question is what properties could be related with the sensitivity of *D* and *λ* on *ρ*.

### Solvation structure

**Figure 4:**
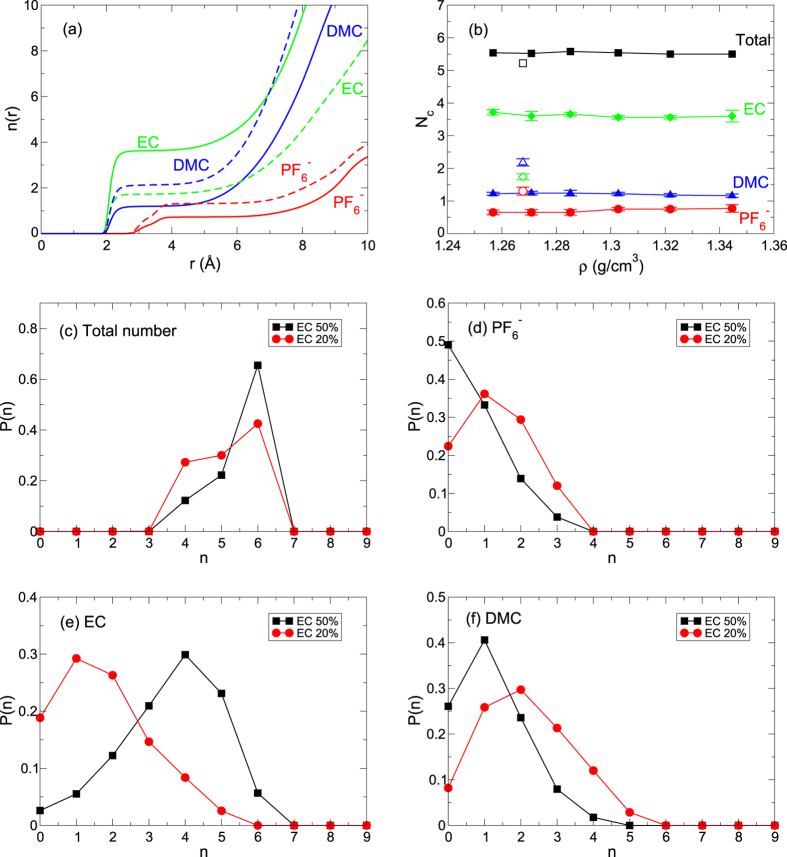
Solvation structure of a Li^+^ ion. (**a**) Cumulative coordination numbers *n*(*r*) of a PF_6_^−^ ion, EC and DMC as a function of distance *r* from a Li^+^ ion at a temperature of *T* = 300 K for solvent mixture ratios of EC 50% at a density *ρ* = 1.3446 g/cm^3^ and EC 20% at a density *ρ* = 1.2677 g/cm^3^. Solid and dashed lines denote the cases of EC 50% and EC 20%, respectively. Note that we calculate *n*(*r*) from the positions of a P atom for a PF_6_^−^ ion and a carbonyl oxygen O atom for both EC and DMC. (**b**) The solvation number *N*_*c*_ in the first solvation shell of a Li^+^ ion as a function of density *ρ* at a temperature of *T* = 300 K. Filled and hollow symbols denote cases of EC 50% and EC 20%, respectively. Next, we present the probability density functions *P*(*n*) of a Li^+^ ion, which represents the probability density for a Li^+^ ion to have *n* neighbors in the first solvation shell for each neighbor of (**c**) the total number, (**d**) a PF_6_^−^ ion, (**e**) EC and (**f**) DMC.

Next we investigate the effects of density on the solvation structure of a Li^+^ ion. We calculate the (cumulative) coordination number *n*(*r*) defined as^11^,^27^,^29^,^30^,^31^,^32^,^33^


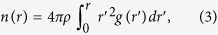


where *g*(*r*) is the radial distribution function (RDF). In Fig. 4(a), we demonstrate *n*(*r*) as a function of a distance *r* from a Li^+^ ion for EC 50% and EC 20%. As shown in Fig. 4(a) and (b), the solvation structure of a Li^+^ ion is much different according to the solvent ratio of EC and DMC^8^,^29^,^30^,^34^. Here we should mention that the graphs of *n*(*r*) for all densities of EC 50% we investigated are almost overlapped to each other, suggesting that the solvation structure of a Li^+^ ion is not affected by the change of *ρ*. In Fig. 4(b), the solvation number *N*_*c*_ in the first solvation shell, defined as the value of *n*(*r*) at the first plateau in Fig. 4(a), keeps a constant as *ρ* varies. The number of each component in the first solvation shell is also the same for all densities we investigated. The small variation in *ρ* does not induce the reorganization of the solvation structure of a Li^+^ ion for a given solvent ratio of electrolytes.

Next we study the probability density function *P*(*n*) for a Li^+^ ion to have *n* neighbors in the first solvation shell. It describes how many Li^+^ ions have *n* neighbors in the solvation sheath. Since *n*(*r*) and *N*_*c*_ are values averaged over the total number of Li^+^ ions, the detailed description of the composition distribution in the solvation sheath is helpful for the better understanding of the solvation structure. In Fig. 4(c–f), we demonstrate *P*(*n*) for neighbors of the total number, a PF_6_^−^ ion, EC and DMC, respectively. We should note that *P* (*n*) shows the same distribution for all densities of EC 50%, whereas it shows a large difference with respect to the change of the EC fraction. For both EC 50% and EC 20%, most of Li^+^ ions have 6 total neighbors in the solvation sheath. Whereas one or two anions are located in the first solvation shell for EC 20%, Li^+^ ions without anions in the shell become the majority for EC 50%. The percentage of Li^+^ ions is the largest for having one or two ECs for EC 20% but four or five ECs for EC 50%. For DMC, *P* (*n*) shows the maximum at two DMCs for EC 20%, whereas the population of Li^+^ ions with one DMC is the majority for EC 50%. Our results exhibit that the solvation structure of a Li^+^ ion is closely dependent on the solvent mixture ratio but is not affected by the change in *ρ*. Thus, the substantial increase in *D* and *λ* upon decreasing *ρ* does not accompany with the change of the solvation structure. It means that one can increase the mobility of electrolytes by adjusting *ρ* without interrupting the solvation structure of a Li^+^ ion.

### Solvation dynamics

**Figure 5:**
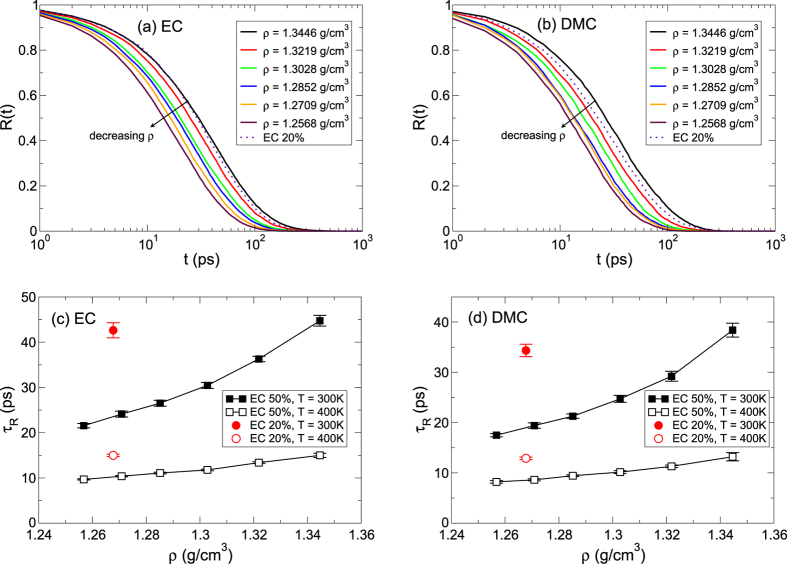
Residence time in a Li^+^ solvation shell. The residence time distributions *R*(*t*) of (**a**) EC and (**b**) DMC within the first solvation shell of a Li^+^ ion at a temperature of *T* = 300 K. Solid lines denote cases of EC 50% for various densities and a dotted line represents a case of EC 20% at a density of *ρ* = 1.2677 g/cm^3^. Next, shown are characteristic residence times *τ*_R_ of (**c**) EC and (**d**) DMC as a function of density *ρ* at temperatures of *T* = 300 K and 400 K. For comparison, we also present *τ*_R_ for EC 20%.

We now study the dynamical properties in the first solvation shell of a Li^+^ ion. The residence time distribution (RTD) *R*(*t*) describes the durability of the first solvation shell of a Li^+^ ion. We define the residence time as the time for an object to escape the first solvation shell of a Li^+^ ion for the first time. Note that from the results of *n*(*r*) in Fig. 4(a), we use the definition of the first solvation shell of a Li^+^ ion as a circle centered at a Li^+^ ion with a radius of 3.0 nm for a carbonyl oxygen of EC and DMC. In Fig. 5(a) and (b), we present the RTDs of EC and DMC at *T* = 300 K for different densities of EC 50%. It clearly shows that the RTD decays faster upon decreasing *ρ* for both solvents. It means that the solvents in the first solvation shell become easier to be replaced by the others for lower *ρ*. Since the solvation structure is independent of the small variation in *ρ*, we presume that the same type of a solvent will replace the pre-existed one. The RTD for EC 50% of densities lower than *ρ* = 1.3446 g/cm^3^ decays faster than one for EC 20% for both EC and DMC, suggesting that the durability of the solvation shell becomes weaker for low *ρ* in EC 50% than EC 20%.

The behaviors of the RTD can be understood by the characteristic residence time *τ*_R_ defined^35^ as


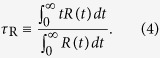


At *T* = 300 K, the characteristic residence times at *ρ* = 1.3446 g/cm^3^ for EC 50% are about 44.7 ps and 38.4 ps for EC and DMC, respectively, and it decreases to 21.5 ps and 17.5 ps upon decreasing density to *ρ* = 1.2568 g/cm^3^, as shown in Fig. 5(c) and (d). At *T* = 400 K, *τ*_R_ decreases from 15.0 ps and 13.2 ps to 9.7 ps and 8.2 ps for EC and DMC, respectively. For both temperatures, *τ*_R_ shows the significant dependence on *ρ*, indicating that the sensitivity of *D* on *ρ* is closely related to the duration of the solvation shell in addition to the activation energy *E*_*a*_ for diffusion.

**Figure 6:**
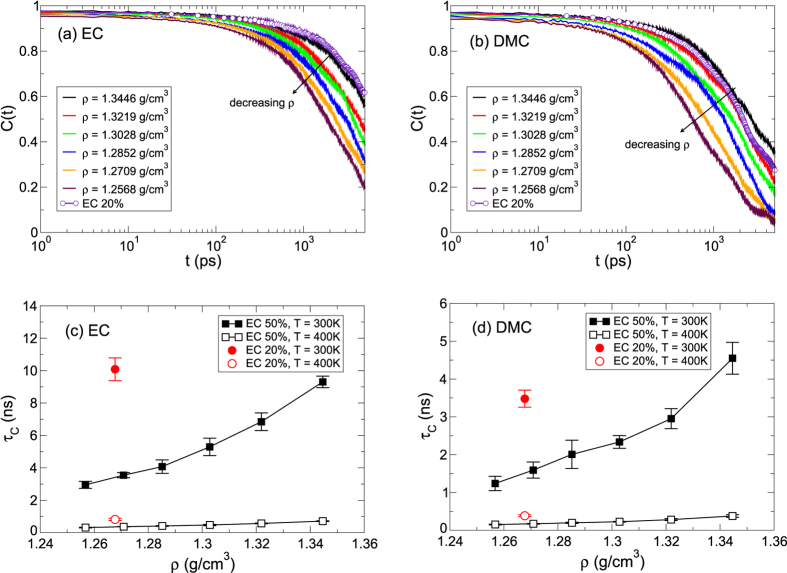
Residence correlation time in a Li^+^ solvation shell. The residence correlation functions *C*(*t*) of (**a**) EC and (**b**) DMC within the first solvation shell of a Li^+^ ion at a temperature of *T* = 300 K. Solid lines denote cases of EC 50% for various densities and a line with circles represents a case of EC 20% at a density of *ρ* = 1.2677 g/cm^3^. Next, shown are characteristic residence correlation times *τ*_*C*_ of (**c**) EC and (**d**) DMC as a function of density *ρ* at temperatures of *T* = 300 K and *T* = 400 K for EC 50%. For comparison, we also present *τ*_*C*_ for EC 20%.

Since the RTD describes the fast kinetics of the solvation dynamics^35^, we now investigate the slow kinetics of the duration of the solvation shell of a Li^+^ ion. To characterize the solvation dynamics in a long time scale, we define the residence correlation function (RCF) *C*(*t*)^17^,^35^ as


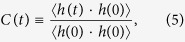


where *h*(*t*) is unity when an object is within the first solvation shell of a Li^+^ ion and *h*(*t*) is zero, otherwise. Whereas the RTD represents the continuous residence time in which the solvent in the solvation shell incessantly remains intact, the RCF describes the discontinuous residence time in the view that the solvent in the solvation shell remains intact only at time *t*, given it was intact at time *t* = 0. In Fig. 6(a) and (b), we present the RCFs of EC and DMC at *T* = 300 K for various densities of EC 50%. The RCF shows the similar behaviors to the RTD with respect to *ρ*. It simply shows that the RCF decays faster for lower *ρ*.

Now we define the residence correlation time *τ*_*C*_ as the time required for *C*(*t*) to decay by a factor of *e*^35^. At *T* = 300 K, the residence correlation time of EC ranges from 3 ns up to 10 ns and for DMC it ranges in the half value of *τ*_*C*_ of EC. At *T* = 300 K, the residence correlation time of EC decreases from *τ*_*C*_ = 9.3 ns to 3.0 ns as density decreases from *ρ* = 1.3446 g/cm^3^ to 1.2568 g/cm^3^. As the temperature increases to *T* = 400 K, *τ*_*C*_ becomes less than 1 ns over the whole density range we investigated. For DMC, the behaviors of *τ*_*C*_ with respect to *ρ* are the same as for EC, even though *τ*_*C*_ of DMC is smaller than *τ*_*C*_ of EC for both temperatures. The decrease in *τ*_*C*_ upon decreasing *ρ* means that the breaking and reforming of the solvation shell of a Li^+^ ion occur more frequently upon decreasing *ρ*. Since the slow kinetics of the solvation dynamics is closely connected with the diffusive dynamics^35^, it indicates that the sensitivity of *D* on *ρ* is related with the durability of the solvation shell.

## Discussion

Increasing the mobility of electrolytes is crucial for the battery performance. A conventional way to increase the mobility at a given temperature has been an increasing fraction of linear carbonates in a binary solvents of electrolytes^17^. However, increasing an amount of linear solvents is limited by the ion-pairing of salts, causing a decrease in the ionic conductivity. Therefore, it has been of great interest to find an optimal ratio of solvent mixtures to give a maximal ionic conductivity. In this aspect, our results suggest that the density of electrolytes can induce a dramatic effect on the dynamics of electrolytes. Even the effects of density can sometimes generate more dramatic results than the solvent mixture ratio.

Our study of the fundamental properties of bulk electrolytes reveals that organic liquid electrolytes consisting of EC and DMC have a larger sensitivity of the diffusive dynamics on density than other liquids^22^,^24^,^36^. Although a small variation in density significantly changes the activation energy for diffusion, it does not induce the reorganization of the solvation structure of a Li^+^ ion. Rather, decreasing density causes the faster solvation dynamics in both short and long time scales. It indicates that breaking and reforming the solvation shell of a Li^+^ ion occur rapidly upon decreasing density. The decrease in density, that is, the increase in the molar volume provides a more room for diffusion and a more chance to interrupt the solvation shell by solvents outside the shell. Solvents binding to a cation are usually one of main reasons to make the system viscous^37^,^38^,^39^. Thus, the frequent reforming of the solvation shell will contribute to enhance the diffusivity. It explains the sensitivity of diffusivity on density.

Even though density can significantly affect the mobility of the system, we would like to mention that it does not follow through with the direct improvement of the battery performance. For example, the transference number, the fraction of the total current carried by a given ionic species, is one of main properties characterizing the efficiency of electrolytes^40^,^41^. In this case, the transference number does not rapidly increase upon decreasing density, since the diffusion constants of both cations and anions increase with a similar rate. Our results, however, will guide the fact that density can play a role in enhancing mobility. Finally, our fundamental study of bulk electrolytes will suggest an intriguing point for understanding and designing electrolytes of lithium-ion batteries.

## Methods

We performed extensive molecular dynamics (MD) simulations of non-aqueous electrolytes of lithium ion batteries consisting of a solution of 1 M LiPF_6_ salt in a binary solvent mixture of EC and DMC. We carried out all simulations using the MD simulation package, LAMMPS^42^. We implemented the OPLS/AA force field to describe the molecular interaction of the solvents. We computed the long-range interactions using particle-particle particle-mesh (PPPM) algorithm. The simulations are performed in the *NVT* ensemble, where *N, V*, and *T* are the number of molecules, the volume, and the temperature, respectively. The linear size of the simulation box ranges from *L* = 5.2672 nm up to 5.3872 nm depending on density. We kept the temperature constant via the Nóse-Hoover thermostat during the simulations. We applied periodic boundary conditions in all three directions of the simulation box. We used 1 fs as a timestep of the simulation.

We investigated the solvent mixture ratios of EC:DMC = 50%:50% and 20%:80% (in vol%). If the two systems have the same volume and one does not consider the mixing effect of the two systems, the final densities of two solvent mixtures based on the individual EC and DMC densities^1^,^5^ are *ρ* = 1.3446 g/cm^3^ and 1.2677 g/cm^3^ (including a LiPF_6_ salt) for EC 50% and EC 20%, respectively. Since the mixed density of binary solvents used in experiments turns out to be lower than the above density^21^, we selected five more cases of lower densities *ρ* = 1.2568, 1.2709, 1.2852, 1.3028, and 1.3219 g/cm^3^ for EC 50% to investigate how the density affects the dynamics of the system and compare with the results of the solvent mixture of EC 20%.

## Additional Information

**How to cite this article**: Han, S. A salient effect of density on the dynamics of nonaqueous electrolytes. *Sci. Rep.* **7**, 46718; doi: 10.1038/srep46718 (2017).

**Publisher's note:** Springer Nature remains neutral with regard to jurisdictional claims in published maps and institutional affiliations.
